# Cancer de la cavité orale chez trois frères germains en Mauritanie

**DOI:** 10.11604/pamj.2016.25.156.10377

**Published:** 2016-11-14

**Authors:** Nacer Dine Baba

**Affiliations:** 1 Service Anatomie Pathologique CHN de Nouakchott, BP 1164 Nouakchott Mauritanie

**Keywords:** Cancers, cavité orale, histoire familiale, Mauritanie, Cancers, oral cavity, family history, Mauritania

## Abstract

Les facteurs de risque majeurs pour les cancers de la cavité orale sont la consommation d'alcool et le tabac mais une composante héréditaire a également été évoquée. L’observation que nous vous présentons ci-dessous a pour objectif d’illustrer cette composante parmi les autres facteurs de risque plus connus. C’est l’histoire de trois frères germains ayant présenté chacun un cancer de la cavité orale avec une évolution très rapide en moins d’une année pour chacun d’entre eux. En plus du facteur familial, les facteurs de risque retrouvés chez ces patients étaient: le tabagisme, une mauvaise hygiène bucco-dentaire, une alimentation pauvre en légumes et en fruits. Le risque familial des cancers de la cavité orale était pendant très longtemps un sujet controversé mais plusieurs études récentes ont suggérées l’existence de ce risque ce qui concorde avec notre observation chez ces trois frères. Ces études ont montré aussi que bien que la composante héréditaire pour les carcinomes des voies aéro-digestives supérieures semble probable, il est important que les membres de la famille à risque comprennent que leur vulnérabilité à ces tumeurs peut être considérablement réduite par l'arrêt du tabac, la modération de la consommation d'alcool et la consommation fréquente de fruits et légumes crus. Des études plus poussées devront être réalisées dans notre pays pour préciser la place respective de ces différents facteurs de risque pour ce cancer. En attendant, la prévention et le diagnostic précoce restent les moyens les plus appropriées pour la lutte contre ce type de cancers.

## Abstract (en)

Major risk factors for cancers of the oral cavity are alcohol and tobacco, but hereditary factors were also identified. This case study aims to illustrate this last component among the most known risk factors. We here report three cases of brothers of the whole blood presenting with cancer of the oral cavity showing rapid development in less than a year in each of them. Additional risk factors other than hereditary factors were: smoking, poor oral hygiene, low fruit and vegetable intake. Hereditary risk has been a controversial issue for a long period of time, but several recent studies have suggested the existence of this risk factor which is consistent with our observation. These studies even showed that hereditary component for carcinomas of the upper aerodigestive tract seems likely; it is important that family members at risk understand that their vulnerability to these tumors can be greatly reduced by stopping smoking, by a moderate consumption of alcohol and a frequent consumption of raw fruits and vegetables. Further studies should be carried out in our country to clarify the respective role of these different risk factors in developing this type of cancer Meanwhile, prevention and early diagnosis are the most appropriate means to fight against this type of cancer.

## Introduction

Les cancers de la cavité orale regroupent l’ensemble des tumeurs malignes développées au niveau de la muqueuse de recouvrement de la bouche et des lèvres. La consommation d'alcool et de tabac sont des facteurs de risque majeurs pour ces cancers [1] mais une composante héréditaire a également été évoquée, bien que seules des données limitées sont disponibles sur cette composante [2]. L’observation que nous vous présentons ci-dessous a pour objectif d’illustrer cette composante parmi les autres facteurs de risque plus connus.

## Patient and observation

C’est l’histoire de trois frères germains ayant présenté chacun un cancer de la cavité orale. Le début remonte à l’an 2000, lorsque M. KM, enseignant de profession, le deuxième de cette fratrie, âgé de 57 ans à cette période, a présenté un carcinome épidermoïde de l’amygdale avec métastases ganglionnaires. Diagnostiqué à Nouakchott, il est évacué au Maroc pour radiothérapie qui n’était pas disponible à Nouakchott à cette période. Après son retour à Nouakchott où il est suivi, l’évolution s’est faite sur 8 mois avec décès dans un tableau d’aphagie totale, d’altération de l’état général et de complications de la radiothérapie sous forme de mucite. Les facteurs de risque retrouvés chez ce patient sont: le tabagisme, une mauvaise hygiène bucco-dentaire avec caries dentaires et enfin une alimentation pauvre en légumes et en fruits. En 2003, M. HM, commerçant, le plus âgé ayant à cette date, 63 ans a présenté un carcinome épidermoïde de la gencive avec métastases ganglionnaires. Après le bilan réalisé, les médecins décident de lui donner un traitement palliatif à base d’antalgiques et d’antibiotiques. L’évolution s’est faite vers une altération progressive de l’état général et le décès après quelques mois. Les mêmes facteurs de risque sont retrouvés chez ce patient en plus du cancer chez son frère En 2012, M. AM, enseignant, âgé de 55 ans a présenté un carcinome épidermoïde de la face interne de la lèvre inférieure diagnostiqué à Nouakchott et évacué en Tunisie, où il a fait l’objet d’une chirurgie première avec chimiothérapie néo-adjuvante. A son retour, apparition d’une grosse adénopathie sous-maxillaire gauche Il est alors orienté au Centre National d’Oncologie de Nouakchott pour des séances de radiothérapie à visée palliative. Il développe alors des complications à type de mucite et décède dans un tableau d’aphagie totale avec cachexie. L’évolution totale s’est faite en une année. On retrouve là aussi les mêmes facteurs de risque avec le cancer chez ses deux frères.

## Discussion

Le risque familial des cancers de la cavité orale était pendant très longtemps un sujet controversé. Mais plusieurs études récentes ont suggérées l’existence de ce risque ce qui concorde avec notre observation chez ces trois frères. En effet, une étude cas-témoins menée à Porto Rico [3] dont l'objectif était de déterminer si les patients atteints de cancers de la cavité orale étaient plus susceptibles que les témoins d'avoir un parent ou un frère avec une histoire déclarée de cancer de la cavité buccale ou des voies aérodigestives supérieures, a trouvé qu’il y a un risque 2,5 fois plus élevé chez les sujets ayant déclaré avoir un parent au premier degré atteint de cancer de la cavité buccale et un risque 2,6 fois plus élevé s’ils ont un parent de premier degré atteint de cancer des VADS. Cette étude a révélée une tendance familiale aux cancers de la cavité buccale et à d’autres cancers des VADS mais, comme pour notre observation, les auteurs ont signalé qu’il est difficile de préciser la part respective des effets de prédisposition héréditaire, du mode de vie partagé ou une combinaison de ces facteurs. Dans une autre étude cas-témoins sur les cancers de la bouche, du pharynx, et du larynx dans le sud du Brésil [4], les auteurs ont trouvé un risque significativement plus élevé associé à une histoire familiale de carcinome des VADS. Une composante héréditaire de prédisposition au cancer oral a été suggérée par des rapports de cas de familles avec plusieurs membres atteints [5], par les études épidémiologiques indiquant une tendance familiale au cancer oral ou d'autres cancers de la tête et du cou [6–8]. Une personne risque davantage d’avoir un carcinome épidermoïde dans la région de la tête et du cou (qui comprend la cavité buccale) si un membre au premier degré de sa famille (père ou mère, frère ou sœur, enfant) a reçu un diagnostic de carcinome épidermoïde à la tête ou au cou. Ces études ont montré aussi que bien que la composante héréditaire pour les carcinomes des VADS semble probable, il est important que les membres de la famille à risque comprennent que leur vulnérabilité à ces tumeurs peut être considérablement réduite par l'arrêt du tabac, la modération de la consommation d'alcool et la consommation fréquente de fruits et légumes crus. Il existe aussi une susceptibilité génétique pour ce type de cancer comme le montrent d’autres études. Des études de la cavité buccale et les carcinomes des VADS ont rapporté une plus grande sensibilité mutagène chez les patients ayant des antécédents familiaux de cancer [9]. Ces observations suggèrent le rôle de l'instabilité génétique comme un facteur de risque pour les carcinomes des VADS et de la cavité orale. Le risque associé à la sensibilité mutagène semble être indépendante de l’âge, du sexe, de l'alcool, du tabac et du régime.

## Conclusion

Notre observation suggère l’existence de formes familiales de cancers de la cavité orale dans notre pays bien que d’autres facteurs comme le tabac, la mauvaise hygiène bucco-dentaire et la faible consommation de fruits et de légumes peuvent être partiellement incriminés. Ces résultats sont compatibles avec les données de la littérature. Des études plus poussées devront cependant être réalisées pour préciser la place respective de ces différents facteurs de risque de ce cancer dans notre pays en vu d’une prévention et d’un diagnostic précoce qui restent les meilleurs moyens de lutte.
